# Successful Surgical Treatment of a Giant Intraventricular Meningioma: A Case Report and Literature Review

**DOI:** 10.3390/medicina60040560

**Published:** 2024-03-29

**Authors:** Corneliu Toader, Razvan-Adrian Covache-Busuioc, Bogdan-Gabriel Bratu, Luca Andrei Glavan, Andrei Adrian Popa, Alexandru Vlad Ciurea

**Affiliations:** 1 Department of Neurosurgery “Carol Davila”, University of Medicine and Pharmacy, 020021 Bucharest, Romania; corneliu.toader@umfcd.ro (C.T.); bogdan.bratu@stud.umfcd.ro (B.-G.B.); luca-andrei.glavan0720@stud.umfcd.ro (L.A.G.); andreiadrianpopa@stud.umfcd.ro (A.A.P.); prof.avciurea@gmail.com (A.V.C.); 2 Department of Neurosurgery, National Institute of Neurology and Neurovascular Diseases, 077160 Bucharest, Romania; 3 Department of Neurosurgery, Sanador Clinical Hospital, 010991 Bucharest, Romania

**Keywords:** gross total resection, hemiparesis, lateral ventricle, giant meningioma, intraventricular meningioma

## Abstract

In our study, we document the case of a 48-year-old patient who presented at our clinic with various neurological disturbances. Magnetic Resonance Imaging revealed the presence of an intraventricular meningioma located in the body of the left lateral ventricle measuring 60 mm in diameter. This tumor was classified as a giant meningioma, accompanied by a significant amount of digitiform-type edema. A surgical procedure was conducted, resulting in a gross total resection of the tumor. Histopathological analysis identified the tumor as a fibrous meningioma. Postoperative assessments, as well as follow-ups conducted at 3 months and 1 year post-surgery, indicated considerable neurological improvement. The patient exhibited a remission of hemiparesis and gait disturbances along with a marginal improvement in the status of expressive aphasia. This case report underscores the significance of achieving total and safe resection of the tumor and includes an analysis of various cases from the literature, particularly focusing on those that describe minimally invasive surgical approaches and highlight the benefits of radiosurgery in the treatment of giant intraventricular meningiomas.

## 1. Introduction

Meningiomas, classified as the most common type of benign tumors within the skull, constitute 39.2% of all primary tumors in the central nervous system (CNS), as established by [1]. Among these, intraventricular meningiomas (IVMs) represent a smaller portion, accounting for 0.5–3% of all meningiomas, yet they make up a significant proportion (10–15%) of adult intraventricular tumors. Their significance is heightened due to their rarity outside of childhood. IVMs predominantly arise from arachnoid cap cells located in the choroid plexus, with the majority (80%) developing in the lateral ventricle’s atrium or trigone area, followed by occurrences in the third (15%) and fourth ventricles (5%) [2].

A comprehensive review of 625 cases of IVM highlighted their lower tendency for recurrence compared to extra-axial meningiomas, as well as the recurrence rates for grade I (2% versus 7–25%), grade II (14% versus 29–52%), and grade III (31% versus 50–94%) tumors [3]. The mortality rate associated with IVMs is 4%, primarily due to postoperative complications, which account for 65% of these cases. These complications include surgical site hematomas, tumor hemorrhage, infections, pulmonary embolism, bronchopneumonia, and abscess formation, rather than being due to tumor progression or recurrence [4].

In the pediatric population, IVMs show a higher occurrence rate, being identified in about 20% of pediatric meningioma cases, mostly in the age group of 12−14 years [5,6].

IVMs are subject to detailed histological analysis, leading to their categorization into various subtypes based on their cellular morphology. Although there is no universally accepted classification system, significant research efforts like the study by Pereira et al., have sought to establish histopathological patterns. This study, analyzing 681 tumors from 98 publications, found that the vast majority (89.8%) of these tumors were classified as grade I. Identified subtypes included fibrous (39.7%, n = 171), transitional (22.0%, n = 95), meningothelial (18.6%, n = 80), angiomatosus (3.2%, n = 14), psammomatous (2.6%, n = 11), among others (13.9%, n = 60). Additionally, this research identified 7.4% of cases (n = 45) as grade II tumors and 2.8% (n = 17) as grade III tumors [3].

Large meningiomas originating from the intraventricular area are extremely rare. These tumors are typically identified through cerebral enhanced Magnetic Resonance Imaging, presenting as large spherical masses within the ventricles, often exceeding 5 cm in diameter [7]. These large meningiomas usually involve critical areas of the brain and are closely intertwined with neurovascular structures [8]. Surgical removal of these tumors is particularly challenging due to issues like limited operative visibility, increased cerebral edema, heightened vascularization, and the need for more extensive craniotomies [8]. Furthermore, the incidence of peritumoral edema is found to be higher in patients with these larger meningiomas compared to those with smaller tumors [9].

This case report enhances the existing knowledge on the surgical management of giant intraventricular meningiomas, supporting the integration of minimally invasive techniques, the utility of radiosurgery, and the importance of a multidisciplinary approach in achieving optimal patient outcomes. It contributes to clinical practice by offering insights into the complexities of treating large brain tumors and the potential for neurological recovery post-intervention. The successful gross total resection of a giant meningioma, measuring 60 mm in diameter, provides a valuable example of the feasibility and effectiveness of surgical intervention in such challenging cases, highlighting the potential for considerable neurological improvement post-surgery, as evidenced by the remission of hemiparesis and gait disturbances, along with improvement in expressive aphasia in the patient.

## 2. Materials and Methods

We report a rare case of a giant intraventricular meningioma in a 48-year-old female, admitted in 2021 to the Department of Neurosurgery, National Institute of Neurology and Neurovascular Diseases, Bucharest, Romania. After a complete surgical resection of the tumoral process, a patient clinico-radiological evaluation was undergone for a period of 1 year, with no tumoral recurrences observed and only a mild expressive aphasia being present.

To deepen the current knowledge of this complex neurosurgical pathology, a comprehensive review has been performed, comparing our case with others from the literature and navigating through different therapies beyond surgery for such cases. The database used was PubMed Central, and, in regard to search formula, the following terms were used: “intraventricular meningioma”, “giant intraventricular meningioma”, “histopathology”, “gross tumor resection”, “neurosurgical approaches”, “gamma knife radiosurgery”, “postoperative complications”. Only case reports, case series or retrospective studies in English from the last 20 years have been included in our study, all at least including the terms “intraventricular meningioma” or “giant intraventricular meningioma”.

## 3. Case Presentation

A 48-year-old female patient, recently diagnosed with an intra-axial tumor in the left cerebral hemisphere, was admitted to our clinic for advanced diagnostic procedures. Neurological evaluation indicated disturbances in balance and gait, symptoms of expressive aphasia, and a right-sided hemiparesis with a Medical Research Council (MRC) strength rating of 4/5.

**Figure 1:**
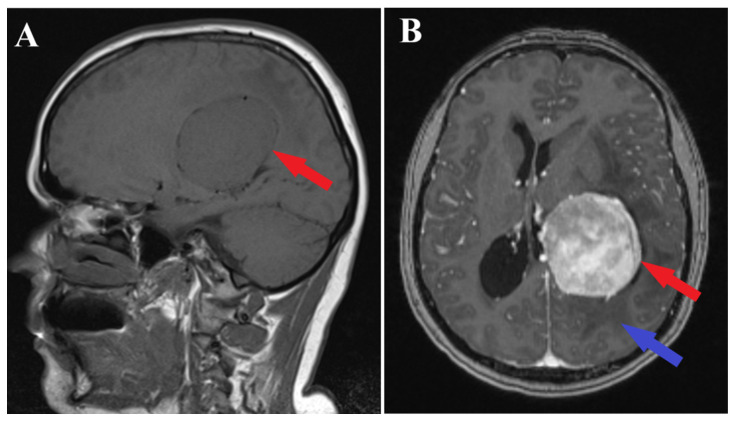
Preoperative MRI T1 and T1Gd sequence. Sagittal section of MRI T1 sequence (**A**) and axial section of MRI T1 Gd sequence (**B**), depicts an intraventricular mass into the left ventricle body (red arrows) with associated perilesional edema (blue arrow).

**Figure 2:**
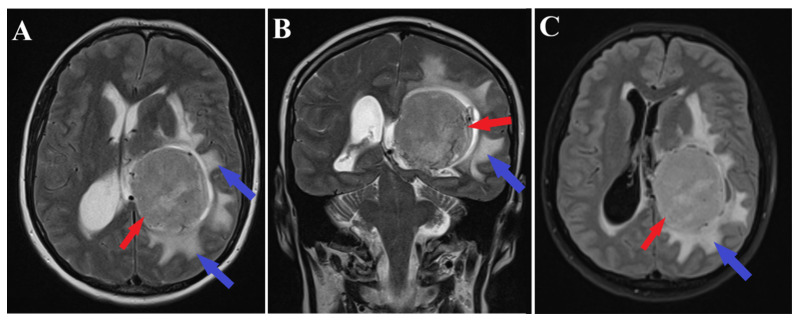
Preoperative MRI T2 and T2 FLAIR sequence. Axial section of MRI T2 sequence (**A**), coronal section of MRI T2 sequence (**B**) and axial section of T2 FLAIR sequence (**C**) are shown. Arrows indicate the intraventricular meningioma (red arrows) and significant digitiform edema (blue arrows).

A brain MRI (i.e., multi-planar, multi-sequential brain MRI examination was performed on a General Electric 3T Signa Architect, Chicago, IL, USA) with contrast enhancement revealed an intraventricular tumor located in the body of the left lateral ventricle. The tumor presented as an ovoid, well-delineated mass with T1 hypointensity and T2 hyperintensity, measuring up to 60 mm in maximum diameter. It induced deformation of the left lateral ventricle. The tumor was associated with substantial digitiform edema, causing a shift in the midline structures and a potential risk of subfalcine herniation (Figure 1 and Figure 2).

**Figure 3:**
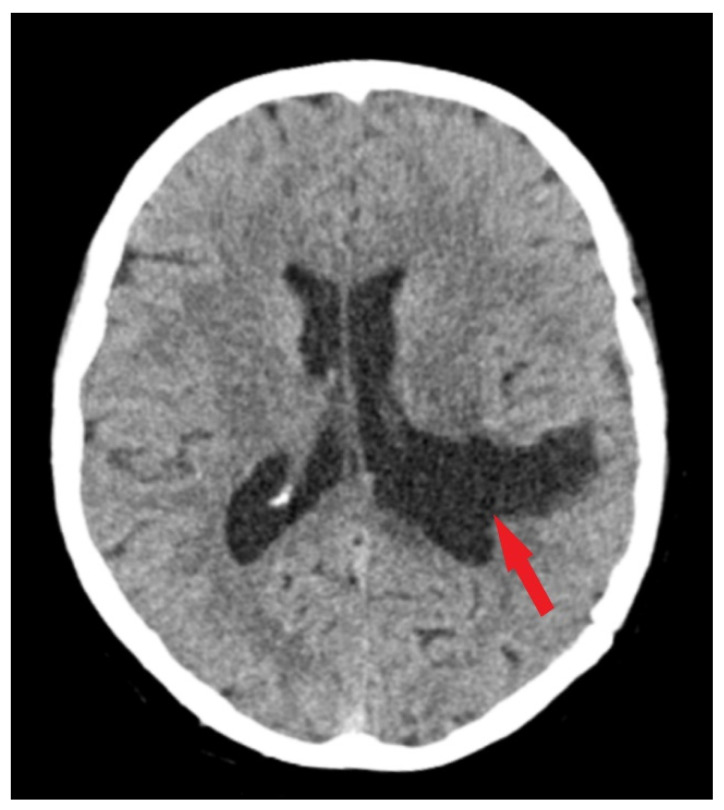
Postoperative CT scan. Tissue window of CT scan highlights total removal of the intraventricular meningioma (red arrow) with edema remission.

**Figure 4:**
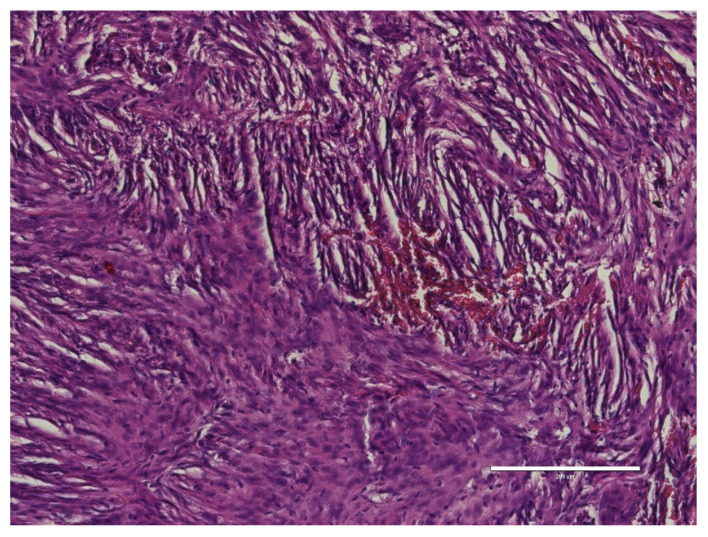
Histopathological examination. Image depicts the characteristic aspect of an fibrous meningioma with multiple spindle cells present, 200×.

Under general anesthesia, a surgical procedure involving a left temporoparietal craniotomy was executed. This was followed by the excision of an intraventricular meningioma situated in the body of the left lateral ventricle under microscopic guidance. We have used this approach in order to exclude the risk of a seizure disorder. Complete resection of the tumor was accomplished (Figure 3), and subsequent histopathological analysis confirmed the tumor as a fibrous meningioma (Figure 4).

This positive outcome reflects a substantial recovery trajectory post-surgery, underscoring both the effectiveness of the medical intervention and the patient’s resilience. The persistence of expressive aphasia, although improved, suggests that the patient might benefit from ongoing speech and language therapy to further enhance communication abilities. Such therapeutic interventions are crucial for improving quality of life and facilitating more effective communication, both of which are essential for the patient’s social integration and psychological well-being. The complete recovery of muscle strength in the right side, reaching an MRC score of 5/5, is particularly encouraging. This score signifies that the patient can overcome resistance with full power in the previously affected limbs, a testament to the successful rehabilitation efforts and the patient’s determination in physical therapy. It is important for the patient to continue with physical and rehabilitative exercises to maintain this muscle strength and to prevent any potential complications or decline in physical capabilities.

Overall, the patient’s postoperative evolution into the discharge phase paints a picture of significant neurological recovery, albeit with remaining challenges. The medical team’s focus going forward will likely involve a multidisciplinary approach, incorporating continued physical therapy, targeted speech and language therapy, and regular neurological assessments to monitor progress and address any emerging issues. This comprehensive care plan is crucial for supporting the patient’s journey towards full recovery and optimizing their functional independence and quality of life.

**Figure 5:**
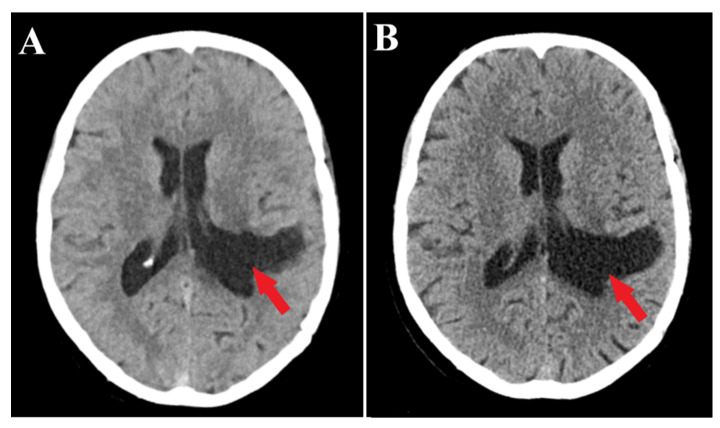
Follow-up CT scans. 3 months follow-up CT scan (**A**) and 1 year follow-up CT scan (**B**) demonstrate successful complete resection of the meningioma (red arrows) without residual tumor fragments.

Follow-up evaluations at 3 months and 1 year post-surgery revealed mild, predominantly expressive mixed aphasia. Non-contrast CT scans identified a region of hypodensity in the left temporo-parietal area, indicative of post-surgical changes without any mass effect or contrast uptake, suggesting a sequela. No evidence of tumor recurrence or residual meningioma was observed (Figure 5). Throughout the follow-up period, the patient was maintained on antiepileptic medication, with no reported episodes of seizures.

## 4. Discussion

Fibrous meningiomas, also known as fibroblastic meningiomas, are characterized by a significant presence of mesenchymal components, including gelatinous fiber, bone, and myxoid tissue, which can lead to the calcification or ossification of the tumor. Within the category of solitary fibrous tumors, the World Health Organization (WHO) generally assigns a grade I classification to lesions marked by high collagen content and low cellularity, typically identified as solitary fibrous tumors [10].

Meningiomas are also classified based on their size. According to this classification, a meningioma is labeled as ‘giant’ if it measures over 5 cm in diameter, aligning with WHO grade III criteria [11]. In certain cases, such as the one being discussed, this size threshold is exceeded; however, the precise biological mechanisms that enable meningiomas to grow to these ‘giant’ sizes remain largely unknown.

The primary treatment for giant intraventricular meningiomas is gross total resection, which is often curative. In cases where achieving vascular control is challenging, a combined approach of partial resection followed by adjuvant radiosurgery is considered a viable and safe option. Needle biopsy may be utilized for diagnostic purposes in patients who are not candidates for surgical treatment, followed by radiosurgery. In cases where the diagnosis is clear, radiosurgery alone may suffice [12]. The choice of surgical technique depends on various factors, including the tumor’s specific location within the ventricle, its side, and its size [13,14].

In a comprehensive study of 89 cases, preoperative contrast-enhanced magnetic resonance imaging (MRI) scans were conducted for all patients. The distribution of intraventricular meningiomas across different ventricles was as follows: 70 cases (79%) in the lateral ventricle, 12 cases (13%) in the third ventricle, and 7 cases (8%) in the fourth ventricle. Notably, there was a higher occurrence of lateral ventricular meningiomas on the left side compared to the right, with 41 instances on the left and 29 on the right. The majority of these tumors were located in the trigone area of the lateral ventricle, while tumors in the frontal horn, temporal horn, or the body of the ventricle were less common.

The study also noted a tendency for meningiomas in the lateral ventricle to be larger than those in other ventricular locations. However, this finding did not reach statistical significance, with a *p*-value of 0.07 [15].

The accompanying table summarizes various studies detailing the common characteristics of giant intraventricular meningiomas (GIVMs). A recurrent histological feature in GIVMs is the fibromatous type, which is well-supported by the existing literature. Additionally, an analysis of diverse cases shows that the most frequent anatomical site for GIVMs is the lateral ventricle and its subdivisions, a pattern also reflected in the case study being discussed, emphasizing its significance in this medical context (Table 1).

In the realm of treatment strategies for giant intraventricular meningiomas (GIVMs), the use of minimally invasive surgical techniques has shown promising outcomes. A notable instance is the study by Jamshidi et al., which documented the successful treatment of three patients with GIVMs located in the lateral ventricle [18]. These patients underwent surgery using the parietal approach port technique, achieving thorough tumor removals. Post-surgical outcomes were favorable, with patients demonstrating positive recovery and no reports of tumor recurrence, thus positioning this study as a pioneering example of GIVM removal using port techniques.

When addressing GIVMs in the atrium of the lateral ventricle, a comprehensive understanding of the surrounding anatomy is crucial for selecting the most appropriate surgical pathway. This involves assessing anterior, posterior, and lateral surgical approaches to the atrium [20]. The anterior approach, particularly on the left side, poses risks to the auditory cortex. In contrast, posterior approaches may lead to disconnection syndromes, such as alien hand syndrome, especially in transversal surgeries across the right and left hemispheres. Temporal transcortical approaches are generally preferred for lesions in the non-dominant hemisphere to preserve language areas. Nonetheless, navigating through the middle and inferior temporal gyri might endanger the visual field due to the optic radiation fibers located near the atrium and temporal horn [20]. Recent developments indicate that a transcortical route through the left or right middle temporal gyrus could reduce postoperative complications [18].

Tubular retractors are emerging as an innovative alternative to traditional blade retractors in creating a surgical corridor during intracranial procedures. In contrast to paddle retractors, which apply pressure at a single point, tubular retractors distribute this pressure evenly across their circumference. This feature is believed to reduce the risk of localized trauma, thereby lessening the chances of vascular damage and the risk of ischemia. The current market offers a variety of tubular retractor systems, each with distinct material properties, flexibility, and designs. These systems have been employed in the removal of various intracranial tumors, including colloid cysts, deep-seated gliomas, and cavernous hemangiomas. However, most existing research consists of small-scale studies conducted by single surgeons, primarily focused on the resection of certain lesion types. This narrow scope may hinder the recognition of less frequent complications, such as seizure risks associated with a transcortical approach. Consequently, there is a noticeable gap in comprehensive data on the use of tubular retractors across diverse patient groups and among surgeons with varying levels of experience [21,22,23,24,25].

Despite these limitations, the use of tubular retractors has been acknowledged for facilitating a minimally invasive approach in the extraction of intracranial lesions, as supported by previous research [26]. This cannot be applied in our case, as we used an open-surgery approach.

The introduction of primary stereotactic radiosurgery (SRS) has significantly altered the treatment approach for intraventricular meningiomas (IVMs), especially considering the challenges posed by their deep location and proximity to critical neurovascular structures [3]. In a particular study series, all patients who underwent follow-up assessments and imaging scans showed consistent local control for an extended period post-SRS. Additional studies focusing on SRS for IVMs have also reported high local control rates, albeit with some variations. For instance, in a study by Kim et al., local control was achieved in 78% (seven out of nine cases) after an average follow-up of over five years [12]. These control rates are in line with those seen in SRS treatments of meningiomas in other brain regions, including in older patients [27].

One of the challenges in comparing local response rates among different IVM-focused studies is the variability in response assessment criteria. To standardize this, the referenced study utilized the Response Assessment in Neuro-Oncology (RANO) criteria, making it the first in IVM research to consistently apply these standards [28]. Additionally, gamma knife radiosurgery (GKRS) has become increasingly used for treating intracranial meningiomas in various locations like the skull base, tentorium, falx, and convexity. However, specific data on the use of GKRS for IVMs remains limited, with the literature review finding only a few case series addressing IVMs treated with radiosurgery.

Postoperative Recommendations: All patients with intracranial hypertension (ICH) should undergo exploration using CT and MRI with a contrast agent, as well as screening for hemorrhage on the second day with a native CT scan, followed by follow-up appointments after discharge at 6 months, 1 year, and 2 years. If there is residual tumor tissue, Gamma Knife radiotherapy (if the size permits), stereotactic radiotherapy, or whole brain radiation therapy may be considered.

## 5. Conclusions

In concluding our analysis of this case involving a giant intraventricular meningioma, we have delineated key aspects of this condition, correlating our observations with current research in the field. Notably, this case displays characteristics typical of fibromatous meningiomas in terms of histology, and the tumor’s primary location in the lateral ventricle aligns with findings from other studies on giant intraventricular meningiomas (GIVMs). Our examination of the unique attributes of minimally invasive resection techniques has highlighted their efficacy in tumor removal, with approaches like gross total resection and transcortical temporal routes proving particularly effective.

We have also provided deeper insights into the treatment of these complex tumors, shedding light on both surgical and immunohistochemical approaches. The burgeoning field of molecular targeted therapies has show the potential to offer more precise treatment options for meningiomas. Despite significant progress in the neuro-oncology sector globally, conditions like GIVMs continue to present substantial challenges. Addressing these formidable issues requires a collaborative effort, capitalizing on advancements in both research and clinical practices to transform treatment methodologies and improve patient outcomes in this area.

## Figures and Tables

**Table 1:** Illustrates a compilation of studies about giant intraventricular meningiomas (GIVM) and their features.

First Autor and Year	N	Histological Aspect	Tumor Location			Extent of Removal N (%)		Surgical Approach
			Lateral Ventricles	Third Ventricle	Forth Ventricle	GTR	STR	
Nakamura et al., 2003 [16]	7	Fibromatous type;	6 (85.71%)	1 (14.28%)	0 (0%)	7 (100%)	0 (0%)	Transcortical parieto-occipital approach (6 patients; 85.71%) Transcallosal approach (1 patient; 14.28%)
Liu et al., 2013 [17]	1	NA	1 (100%)	0 (0%)	0 (0%)	1 (100%)	0 (0%)	Microsurgical resection of the left atrium via a transcortical parieto-occipital approach
Chen et al., 2019 [15]	89	WHO Gr I: 72 patients; WHO Gr II: 17 patients.	70 (78.65%)	12 (13.48%)	7 (7.86%)	75 (84.26%)	14 (15.73%)	Superior parietal lobule approach (43 patients); Temporal approach (24 patients); Transcallosal approach (7 patients); Frontal approach (4 patients); Infratentorial supracerebellar (4 patients); Median suboccipital approach (7 patients);
Jamshidi et al., 2021 [18]	3	Fibromatous aspect: 2 patients.	3 (100%)	0 (0%)	0 (0%)	3 (100%)	0 (0%)	Parietal transcortical approach
Yu et al., 2023 [19]	2	Fibroblastic aspect: 1 patient; Atypical aspect: 1 patient.	2 (100%)	0 (0%)	0 (0%)	1 (100%)	0 (0%)	Microsurgical resection via transtemporoparietal occipital approach

N = number of patients with GIVM; GTR = total growth resection; STR = subtotal growth resection; WHO Gr = histological grade according to the World Health Organization; R = recurrences rate; NA = not available.

## Data Availability

All data is available online on libraries such as PubMed.
